# Molecular Characteristics, Clinical Implication, and Cancer Immunity Interactions of Pyroptosis-Related Genes in Breast Cancer

**DOI:** 10.3389/fmed.2021.702638

**Published:** 2021-09-13

**Authors:** Dandan Xu, Zhipeng Ji, Ling Qiang

**Affiliations:** ^1^Internal Medicine II, The Third Affiliated Hospital of Shandong First Medical University (Affiliated Hospital of Shandong Academy of Medical Sciences), Jinan, China; ^2^Department of Gastrointestinal Surgery, The Second Hospital, Cheeloo College of Medicine, Shandong University, Jinan, China; ^3^Department of Medical Oncology, Shandong Cancer Hospital and Institute, Shandong First Medical University and Shandong Academy of Medical Sciences, Jinan, China

**Keywords:** breast cancer, pyroptosis, signature, prognosis, immune, nomogram

## Abstract

**Objective:** Pyroptosis represents an emerging inflammatory form of programmed cell death. Herein, specific functions and clinical implications of pyroptosis-related genes were systematically characterized in breast cancer.

**Methods:** Expression, somatic mutation and copy number variation of 33 pyroptosis-related genes were assessed in breast cancer from TCGA dataset. Their interactions, biological functions and prognostic values were then observed. By stepwise Cox regression analysis, a pyroptosis-related gene signature was generated. The predictive efficacy in survival was examined by survival analyses, ROCs, univariate and multivariate analyses and subgroup analyses. Associations between risk score (RS) and cancer immunity cycle, *HLA*, immune cell infiltrations, and immune checkpoints were analyzed.

**Results:** Most of pyroptosis-related genes were abnormally expressed in breast cancer. *CASP8, NLRC4, NLRP3, NLRP2, PLCG1, NLRP1, NLRP7, SCAF11, GSDMC*, and *NOD1* occurred somatic mutations as well as most of them had high frequency of CNV. There were closely interactions between them. These genes were distinctly enriched in immune-related processes. A three-gene signature was generated, containing *IL-18, GSDMC*, and *TIRAP*. High RS predicted poorer overall survival, progression, and recurrence. After verification, this RS was an independent and sensitive predictive index. This RS was negatively correlated to cancer immunity cycle. Also, low RS was characterized by high *HLA*, immune cell infiltrations and immune checkpoints. A nomogram including age and RS was generated for accurately predicting 5-, 8-, and 10-year survival probabilities.

**Conclusion:** Pyroptosis-related genes exert key roles in cancer immunity and might be applied as a prognostic factor of breast cancer.

## Introduction

Breast cancer represents a frequently diagnosed malignancy among women globally, with a high mortality (1). This malignancy affects 1/20 globally and 1/8 in high-income countries (2). Females with high risk of developing breast cancer are a heterogeneous population (3). Further research requires to improve prognostic models to stratify high-risk patients. The biology in breast cancer progress is complex in which genetic and environmental elements are involved (4). Conventional breast cancer classification primarily replies on clinicopathologic characteristics and routine markers, not capturing various clinical courses of individual patient (5). In-depth understanding of the molecular mechanisms could lead to improvement in patients' prognoses.

Varied factors are in relation to carcinogenesis, such as activations of proto- and antioncogenes, immune microenvironment as well as chronic inflammation (6, 7). Pyroptosis represents a form of programmed cell death, which may induce the cleavage of *gasdermin D* along with activation of immune and inflammatory response (8). Activated pyroptosis induces the release of the inflammatory factors *IL-1* and *IL-18*, thereby promoting breast cancer initiation. It is featured by cell swelling as well as bubble-like protrusions. Interactions between pyroptosis and cancers are complex due to varied influence of pyroptosis on cancers in distinct tissue specimens as well as genetic background (9). Increasing evidences highlight the roles of pyroptosis in carcinogenesis (10). Previously, increased *GSDMB* expression is related to poor survival and high metastases of breast cancer (11). Furthermore, recent research has demonstrated the crosstalk between pyroptosis and anti-cancer immunity (12). Research has displayed that chemotherapy drugs, miRNAs, etc., may trigger cancer pyroptosis, which inhibits malignant development of cancers (13). Based on pyroptosis regulators, a seven-gene signature has been generated for predicting ovarian cancer prognoses (14). However, no studies have reported prognostic implications of pyroptosis-related gene signature in breast cancer.

Herein, we analyzed molecular characteristics and clinical implication of pyroptosis-related genes as well as their interactions with cancer immunity in breast cancer.

## Materials and Methods

### Data Acquisition

RNA-seq profiling (FPKM values) of breast cancer was retrieved from the Cancer Genome Atlas (TCGA; https://cancergenome.nih.gov/) database. After removing samples without complete clinical information, 1,082 breast cancer samples were included in our study (Supplementary Table 1). Meanwhile, RNA-seq profiles of 113 adjacent normal tissues were also obtained from TCGA database. Then, FPKM values were converted to TPM values according to the following formula: TPMi = FPKMi^*^1000000/(FPKM0 + …. + FPKMm), where i represents gene i and m represents the total number of all genes. Somatic mutation and copy number variation (CNV) data were also retrieved from TCGA database.

### Pyroptosis-Related Genes

We collected 33 pyroptosis-related genes from the published literature, including *GPX4, NLRP7, NLRP2, CASP6, CASP3, TNF, IL1B, IL18, CASP8, NLRP6, IL6, GSDMA, GSDMC, PYCARD, CASP5, AIM2, NOD2, NLRC4, NLRP3, CASP4, CASP1, PRKACA, ELANE, TIRAP, SCAF11, PJVK, CASP9, NOD1, PLCG1, NLRP1, GSDME, GSDMD*, and *GSDMB* (8, 15–17). The location of pyroptosis-related genes on the chromosome was plotted via Rcircos package (18). The mRNA expression of pyroptosis-related genes was compared between breast cancer and normal samples with unpaired student' s *t*-test. Their somatic mutations were analyzed using maftools package (19). Frequencies of genetic amplification and deletion were also summarized. Spearman correlation test was adopted for evaluation of the associations between pyroptosis-related genes across breast cancer samples.

### Protein-Protein Interaction (PPI) and Gene Ontology (GO) Annotation Analysis

Thirty pyroptosis-related genes were uploaded onto the STRING database (https://string-db.org) and their interaction pairs were obtained (20). A PPI network of pyroptosis-related genes was visualized with Cytoscape (https://cytoscape.org/) (21). The gene set of 33 pyroptosis-related genes was analyzed via clusterProfiler package (22). Biological process, cellular component and molecular function of these pyroptosis-related genes were analyzed. Terms with adjusted *p* < *0.05* were significantly enriched.

### Development of a Prognostic Model

To screen which pyroptosis-related genes were related to breast cancer prognoses, univariate analyses were carried out. Genes with *p* < *0.05* were included for conducting a stepwise Cox regression model. Risk score (RS) was calculated by linearly combining regression coefficients multiplied with expression values. Based on the median of RS, patients were stratified into two groups. Overall survival was compared between groups by Kaplan-Meier curves and log-rank tests. Expression patterns of genes in this model were visualized into heatmap. Area under the curve (AUC) of the receiver operating characteristic (ROC) curve was conducted for assessing the predictive efficacy of this model.

### Univariate and Multivariate Cox Regression Analyses

Univariate analyses were applied for estimating the associations between prognoses and age, T, N, M, stage, and RS. Furthermore, multivariate analyses were presented for observing whether these factors were independently predictive of prognoses of breast cancer. Hazard ratio (HR), 95% confidence interval (CI), and *p*-values were separately determined.

### Subgroup Analysis

To investigate the sensitivity of RS in predicting prognoses, patients were stratified into subgroups according to clinical characteristics, including age ≥65 and age <65, M0 and M1, N0 and N1–3, stage I-II and stage III-IV, T1-2 and T3-4 subgroups. OS was compared in high and low RS patients in each subgroup. *P*-values were determined with log-rank tests.

### Assessment of Activated Pathways, HLA, Immune Cell Infiltration, and Immune Checkpoints

Enrichment scores of several cancer-related pathways were estimated by single sample gene set enrichment analysis (ssGSEA) algorithm (23), including IFN-Gamma signature, APM signal, base excision repair, cell cycle, DNA replication, Fanconi anemia pathway, homologous recombination, microRNAs in cancer, mismatch repair, nucleotide excision repair, oocyte meiosis, p53 signaling pathway, progesterone-mediated oocyte maturation, proteasome, pyrimidine metabolism, spliceosome, systemic lupus erythematosus, viral carcinogenesis. The gene sets of above pathways were listed in Supplementary Table 2. Associations between RS and enrichment scores of pathways were then analyzed with Spearman correlation test. Wilcoxon test was applied for estimating the differences in expression of HLA signatures and immune checkpoints between high- and low-risk groups. Also, infiltration levels of 28 immune cells were inferred by ssGSEA algorithm and were compared between groups. Adjusted *p-value* < *0.05* indicated the significant difference between high- and low-risk groups.

### Estimation of Cancer Immunity Cycle

Each step of cancer immunity cycle (24) was inferred with ssGSEA algorithm, as follows: step1: release of cancer cell antigens (IL10, TGFB1, HMGB1, ANXA1, CALR, CXCL10, PDIA3, HSPA1A, HSPA1B, HSPA2, HSPA8, HSPA4, HSPA14, HSPA5, HSPA6, HSPA9, HSPA13, HSPA7, HSPA8, HSPA12A, HSPA12B, HSP90AA1, HSP90AB1, HSP90B1, IFNA2, IFNA1, IFNA13, IFNA6, IFNA21, IFNA4, IFNA8, IFNA5, IFNA7, IFNA14, IFNA16, IFNA10, IFNA17, IFNB1, IFNE, IFNW1), step2: cancer antigen presentation (TNF, IL1A, IL1B, IFNA2, IFNA1, IFNA13, IFNA6, IFNA21, IFNA4, IFNA8, IFNA5, IFNA7, IFNA14, IFNA16, IFNA10, IFNA17, CD40LG, CD40, NT5C, HMGB1, TLR1, TLR2, TLR3, TLR4, TLR5, TLR6, TLR7, TLR8, TLR9, TLR10, HLAA, B2M, TAP1, IL10, IL4, IL13), step3: priming and activation (CD3D, CD3E, CD3G, CD247, CD28, TNFRSF9, TNFSF9, TNFRSF4, TNFSF4, CD27, CD70, TNFRSF14, TNFSF14, CD40, CD40LG, TNFRSF18, TNFSF18, TNFRSF25, TNFSF15, TNFRSF8, TNFSF8, HAVCR1, TIMD4, SLAMF7, SLAMF6, SLAMF1, SLAMF9, SLAMF8, CD2, CD48, CD58, CD226, ICOS, ICOSLG, KLRK1, MICA, MICB, RAET1E, RAET1G, CRTAM, CADM1, CTLA4, PDCD1, PDCD1LG2, CD274, CD160, TNFRSF14, BTLA, VSIR, LAIR1, HAVCR1, HAVCR2, LGALS9, TIMD4, CD244, CD48, TIGIT, NECTIN3, LAG3, IL2, IL12A, IL12B), step4 T cell recruiting (CXCR5, CCR7, CXCL9, CCL3, CCL4, CCL5, CCL19, CCL21, CX3CL1, CXCL13), step4: CD8 T cell recruiting (CCR5, CXCR3, CXCL10, CXCL9, CCL20, CXCL11, CX3CL1, CXCL16), step4: Th1 cell recruiting (CXCR3, CXCL10, CXCL9, CXCL11), step4: dendritic cell recruiting (CCR7, CCL3, CCL4, CCL5, CCL21), step4: Th22 cell recruiting (CCR6, CCL20), step4: macrophage recruiting (CSF1, CCL2, CCL3, CCL4, CCL5), step4: monocyte recruiting (CCL2, CCL7, CX3CL1), step4: neutrophil recruiting (CXCL1, CXCL2, CXCL3, CXCL8, CXCL6, CXCL5), step4: NK cell recruiting (CXCR3, CXCL10, CXCL9, CCL3, CCL4, CCL5, CXCL11, CX3CL1), step4: eosinophil cell recruiting (CCL11, CCL24, CCL26), step4: basophil recruiting (CCL24, CCL26), step4: Th17 cell recruiting (CCR6, CCL20, CXCL12, CXCR4), step4: B cell recruiting (CXCR5, CXCL13), step4: Th2 cell recruiting (CCL1, CCL17, CCL22), step4: Treg cell recruiting (CCR4, CCR10, CCL1, CCL17, CCL22, CCL28), step4: MDSC recruiting (CXCR2, CXCL5), step5: infiltration of immune cells into tumors (STAT1, IRF5, KLF2, ITGB2, ICAM1, EZH2, DNMT1, VEGFA, EDNRB), step6: recognition of cancer cells by T cells (CD28, ICOS, ICOSLG, TNFRSF9, TNFSF9, CD27, CD70, TNFRSF4, TNFSF4, TNFSF14, CD40, CD40LG, HLAA, B2M, TAP1, BIRC5, MDM2, MAGEA4, TP53, PDCD1, PDCD1LG2, CD274, CTLA4, BTLA, VTCN1), step7: killing of cancer cells (IFNG, GZMB, PRF1, PDCD1, SMC3, VTCN1, HAVCR2, MICA, MICB, BTLA, VSIR, LAG3, IDO1, IDO2, ARG1, ARG2, NOS1, NOS2, NOS3, TGFB1, IL10, CCL28, CXCL12, CCL2, CXCL8). Correlation between RS and cancer immunity cycle was assessed.

### Gene Set Enrichment Analysis (GSEA)

GSEA method was employed for identifying enriched Kyoto Encyclopedia of Genes and Genomes (KEGG) in high and low RS groups based on transcriptomic data (25). Terms with nominal enrichment score >2 and false discovery rates <0.05 were significantly enriched.

### Nomogram Construction

Independent prognostic factors were incorporated for constructing a prognostic nomogram in predicting 5-, 8-, and 10-year survival duration with stepwise Cox regression analyses. Calibration plots were plotted for comparing nomogram-predicted and observed 5-, 8-, and 10-year survival.

### Cell Culture

Human normal breast cells (MCF-10A) and breast cancer cells (MDA-MB-231 and HCC70) were retrieved from Shanghai Cell Bank of Chinese Academy of Sciences (China), which were maintained in DMEM plus 10% FBS (Gibco, USA) and 1% penicillin–streptomycin. All cells were incubated at 37°C under the condition of 5% CO_2_.

### Western Blot

MCF-10A, MDA-MB-231, and HCC70 cells were lysed by cell lysates (Beyotime, Beijing, China). Total protein was electrophoresed in 10% polyacrylamide gels. Afterwards, the protein was transferred onto PVDF membrane and blocked with 5% skimmed milk for 2 h at 37 °C. The membrane was incubated by primary antibodies against GSDMC (1:1000; #PA5-101660; Invitrogen, USA), IL-18 (1:1,000; #PA5-19131; Invitrogen, USA), TIRAP (1:1,000; #PA5-88657; Invitrogen, USA), and GAPDH (1:1,000; #ab8245; Abcam, USA) at 4°C overnight, followed by incubation with secondary antibodies (1:2,000; #ab7090; Abcam, USA). Protein bands were acquired through ECL kit (Beyotime, Beijing, China).

### Immunofluorescence

MCF-10A, MDA-MB-231, and HCC70 cells were fixed by 10% formaldehyde for 40 min, followed by being blocked by 5% BSA blocking buffer for 1 h at room temperature. Afterwards, the cells were incubated with GSDMC (1:100; #PA5-101660; Invitrogen, USA), IL-18 (1:100; #PA5-19131; Invitrogen, USA), and TIRAP (1:100; #PA5-88657; Invitrogen, USA) overnight at 4°C. Then, the cells were incubated with secondary antibodies (1:200; ab150077 or ab150080; Abcam, USA) for 30 min at 37°C. The cells were stained by DAPI (Solarbio, Beijing, China) and images were acquired under a fluorescence microscope (Olympus, Japan).

### Statistical Analysis

Data were analyzed with the R (version 3.6.1) and R Bioconductor packages. Comparisons between two groups were evaluated with student' *t*-test or Wilcoxon test. Differences in disease-free interval (DFI), disease-free survival (DFS), disease-specific survival (DSS) and progression-free interval (PFI) were compared in high and low RS groups with Kaplan-Meier curves and log-rank tests. *P* < *0.05* indicated statistical significance.

## Results

### Landscape of Expression and Mutation of Pyroptosis-Related Genes in Breast Cancer

**Figure 1:**
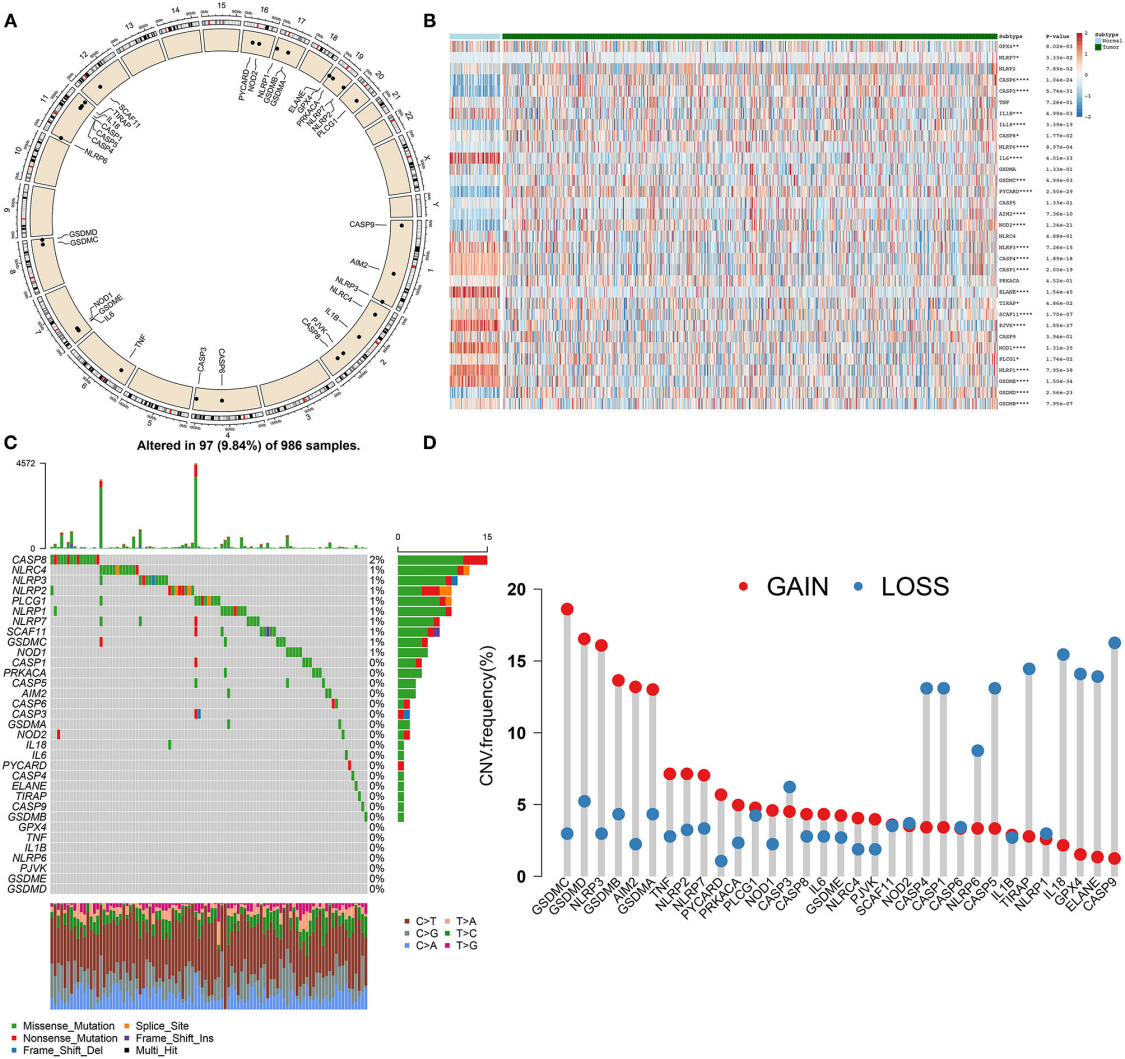
Landscape of expression and genetic mutations of pyroptosis-related genes in breast cancer. **(A)** Circus plots of chromosome distributions of pyroptosis-related genes. **(B)** Heatmap of expression patterns of pyroptosis-related genes in normal and breast cancer tissues. ^*^p < *0.05;* ^**^p < *0.01;* ^***^p < *0.001;* ^****^p < *0.0001*. **(C)** Waterfall chart of somatic mutations of pyroptosis-related genes. **(D)** Frequencies of gain and loss for pyroptosis-related genes.

In this study, 33 pyroptosis-related genes were dissected in breast cancer. Figure 1A displayed the location of these pyroptosis-related genes on chromosomes, as follows: *CASP9* (chr1: 15490832–15526534), *AIM2* (chr1: 159062484–159147096), *NLRP3* (chr1: 247416156–247449108), *NLRC4* (chr2: 32224453–32265854), *IL1B* (chr2: 112829751–112836903), *CASP8* (chr2: 201233443–201287711), *CASP6* (chr4: 109688622–109703583), *CASP3* (chr4: 184627696–184649509), *TNF* (chr6: 31575567–31578336), *IL6* (chr7: 22725884–22732002), *NOD1* (chr7: 3042452–30478784), *GSDMC* (chr8: 129748196–129786888), *GSDMD* (chr8: 143553207–143563062), *NLRP6* (chr11: 278365–285359), *CASP4* (chr11: 104942866–104969436), *CASP5* (chr11: 104994235–105023168), *CASP1* (chr11: 105025443–105035250), *CASP1* (chr11: 105025443–105035250), *IL18* (chr11: 112143251–112164117), *TIRAP* (chr11: 126283065–126298845), *SCAF11* (chr12: 45919131–45992120), *PYCARD* (chr16: 31201485–31203450), *NOD2* (chr16: 50693603–50733077), *NLRP1* (chr17: 5499427–5619424), *GSDMB* (chr17: 39904595–39919854), *GSDMA* (chr17: 39962973–39977766), *PLCG1* (chr20: 41136960–41196801), *ELANE* (chr19: 851014–856247), *GPX4* (chr19: 1103926–1106791), *PRKACA* (chr19: 14091688–14118084), *NLRP7* (chr19: 54923509–54966312), *NLRP2* (chr19: 54953130 – 55001142), *PJVK* (chr2: 179316163–179326117), and *GSDME* (chr7: 24737972–24809244). Expression of above pyroptosis-related genes was compared between breast cancer and normal tissues. Heatmap showed that *GPX4* (*p* = *8.02e-03*), *NLRP7* (*p* = *3.33e-02*), *CASP6* (*p* = *1.04e-24*), *CASP3* (*p* = *5.74e-31*), *IL1B* (*p* = *4.99e-03*), *IL18* (*p* = *3.39e-19*), *CASP8* (*p* = *1.77e-02*), *NLRP6* (*p* = *8.97e-04*), *IL6* (*p* = *4.01e-33*), *GSDMC* (*p* = *4.99e-03*), *PYCARD* (*p* = *2.50e-29*), *AIM2* (*p* = *7.36e-10*), *NOD2* (*p* = *1.34e-21*), *NLRP3* (*p* = *7.26e-15*), and *CASP4* (*p* = *1.89e-18*) possessed aberrant expression patterns in breast cancer than normal tissue samples (Figure 1B). Furthermore, genetic mutations of the pyroptosis-related genes were assessed in depth. In Figure 1C, *CASP8* (2%), *NLRC4* (1%), *NLRP3* (1%), *NLRP2* (1%), *PLCG1* (1%), *NLRP1* (1%), *NLRP7* (1%), *SCAF11* (1%), *GSDMC* (1%), and *NOD1* (1%) occurred genetic mutations in breast cancer. Also, most of pyroptosis-related genes had high frequencies of CNVs in breast cancer (Figure 1D). We further ascertained whether the above genetic mutations affected the expression of pyroptosis-related genes in breast cancer. We observed that CNV could be a dominating factor leading to perturbations on the expression of pyroptosis-related genes. In comparison to normal breast tissues, pyroptosis-related genes with amplificated CNV had distinctly higher expression in breast cancer tissues (such as *GSDMC, GSDMD*, and *AIM2*), and vice versa (such as *CASP4, CASP1*, and *ELANE*).

### Interactions Between Pyroptosis-Related Genes and Their Biological Implications

**Figure 2:**
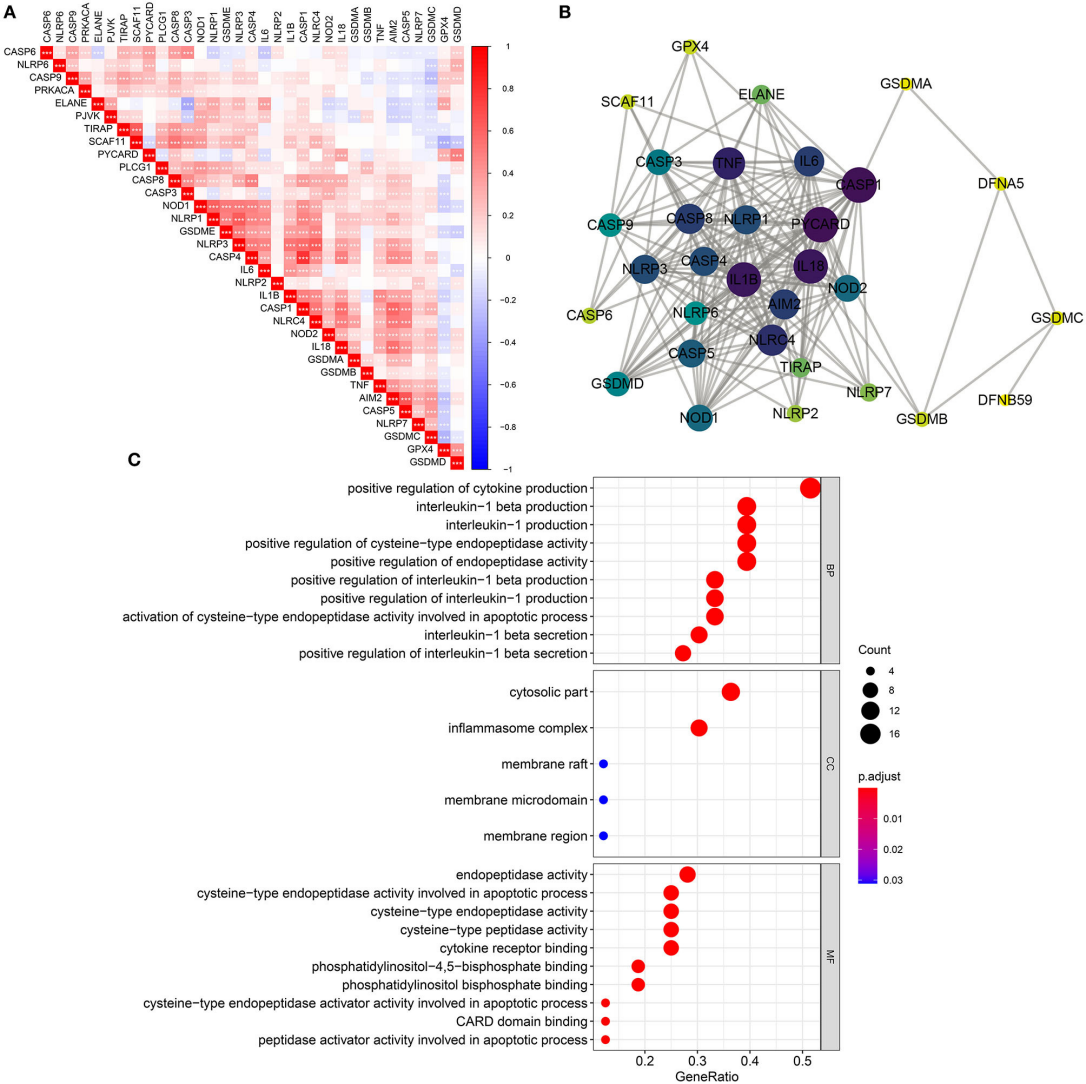
Biological functions and interactions of pyroptosis-related genes in breast cancer. **(A)** Correlations between pyroptosis-related genes. ^*^p < *0.05;* ^**^p < *0.01;* ^***^p < *0.001*. The darker the color, the stronger the correlation. **(B)** A PPI network of pyroptosis-related genes. The larger the circle, the greater the degree. **(C)** Biological process (BP), cellular component (CC), and molecular function (MF) enrichment analyses results of pyroptosis-related genes.

Spearman test was utilized for evaluating correlations between 33 pyroptosis-related genes in breast cancer from TCGA dataset. In Figure 2A, there were tight interactions between them, such as *CASP4* and *CASP1*. A PPI network was than constructed based on 33 pyroptosis-related genes (Figure 2B). Among all nodes, *CASP1* had the highest degree. Furthermore, biological implications of 33 pyroptosis-related genes were evaluated by clusterProfiler package. Our data showed that these pyroptosis-related genes were mainly involved in regulating *IL-1*β production and secretion biological processes (Figure 2C). Furthermore, they participated in cytosolic, inflammasome and membrane cellular components as well as apoptotic process.

### Generation of a Pyroptosis-Related Gene Signature for Breast Cancer Prognoses

**Figure 3:**
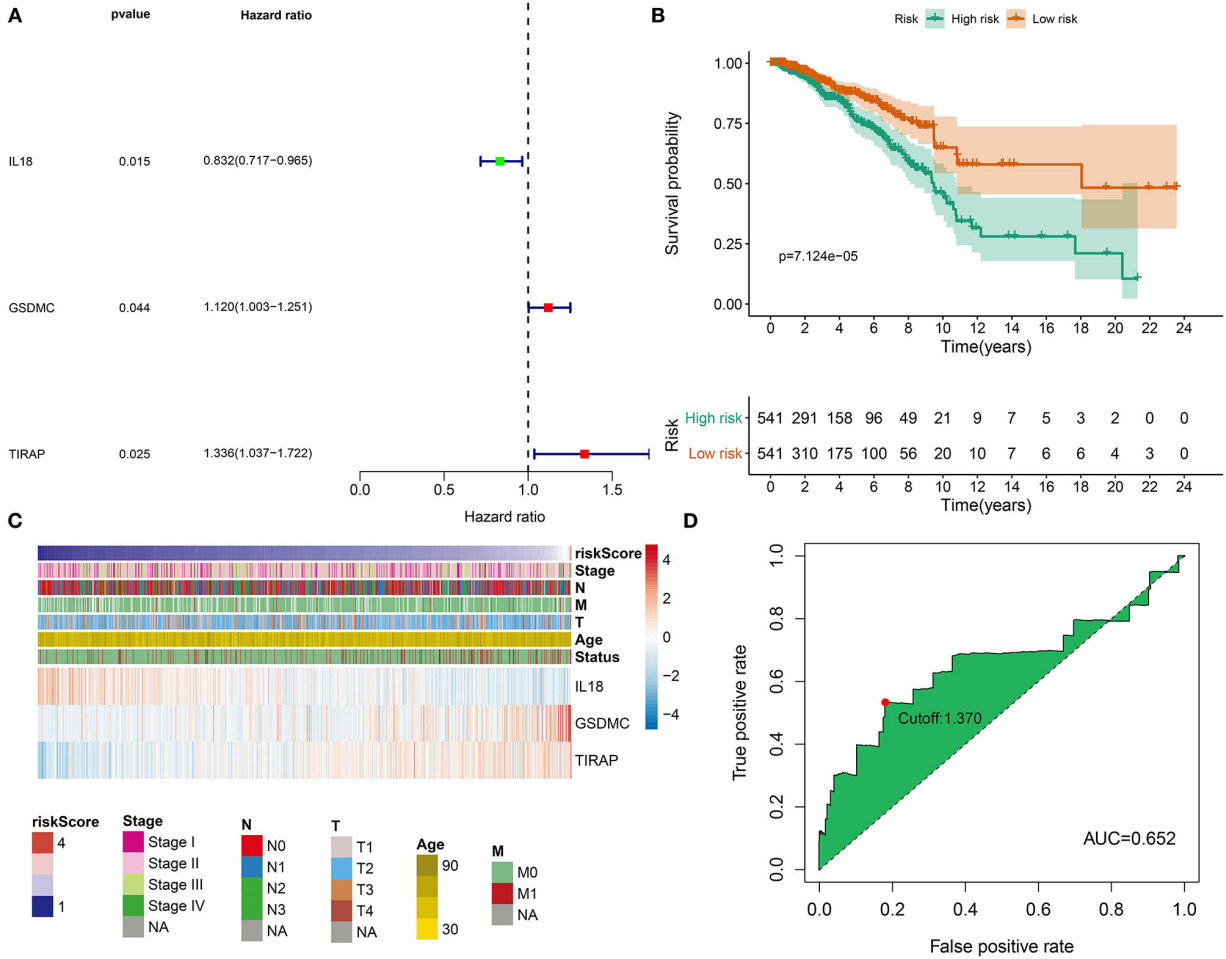
Development of a pyroptosis-related gene signature for breast cancer prognoses. **(A)** Univariate analyses were utilized for screening prognoses-related pyroptosis genes and a pyroptosis-related gene signature was established. **(B)** OS probabilities in high- and low-risk patients, followed by log-rank tests. **(C)** Distributions of *IL18, GSDMC*, and *TIRAP* expression in risk scores and different clinical features. **(D)** ROC curves were plotted for examining the predictive efficacy of this signature.

Clinical implications of pyroptosis-related genes were assessed in breast cancer. As shown in univariate cox regression analyses, *IL-18, GSDMC*, and *TIRAP* were significantly associated with survival outcomes of breast cancer. Among them, *IL-18* [*p* = *0.015*, HR(95%CI): 0.832(0.717–0.965)] was a protective factor as well as *GSDMC* [*p* = *0.044*, HR(95%CI): 1.120(1.003–1.251)] and *TIRAP* [*p* = *0.025*, HR(95%CI): 1.336(1.037–1.722)] were risk factors for breast cancer prognoses (Figure 3A). According to their coefficients and expression levels, we calculated risk score (RS) for each patient (Table 1). Then, we evaluated association between RS and survival outcomes. In Figure 3B, patients with high RS exhibited poorer OS than those with low RS (*p* = *7.124e-05*). Furthermore, we observed expression patterns of *IL-18, GSDMC*, and *TIRAP* in high and low RS samples. As a result, *IL-18* was down-regulated as well as *GSDMC* and *TIRAP* were up-regulated in high RS group compared with low RS group (Figure 3C). We also showed their expression in different clinical features including stage, age, T, N, and M. Time-dependent ROC curves were plotted for verifying the predictive performance. In Figure 3D, AUC was 0.652, indicating that RS possessed high accuracy and sensitivity in predicting prognoses of breast cancer.

**Table 1:** Regression coefficients of genes in the prognostic model.

**Genes**	**Coefficients**	**HR**	**HR.95L**	**HR.95H**	***P***
*IL18*	−0.25528	0.774702	0.662455	0.905969	0.001391
*GSDMC*	0.171771	1.187405	1.061616	1.328099	0.002643
*TIRAP*	0.374042	1.453599	1.122064	1.883091	0.004627

### Independency of the Pyroptosis-Related Gene Signature in Predicting Breast Cancer Prognoses

**Figure 4:**
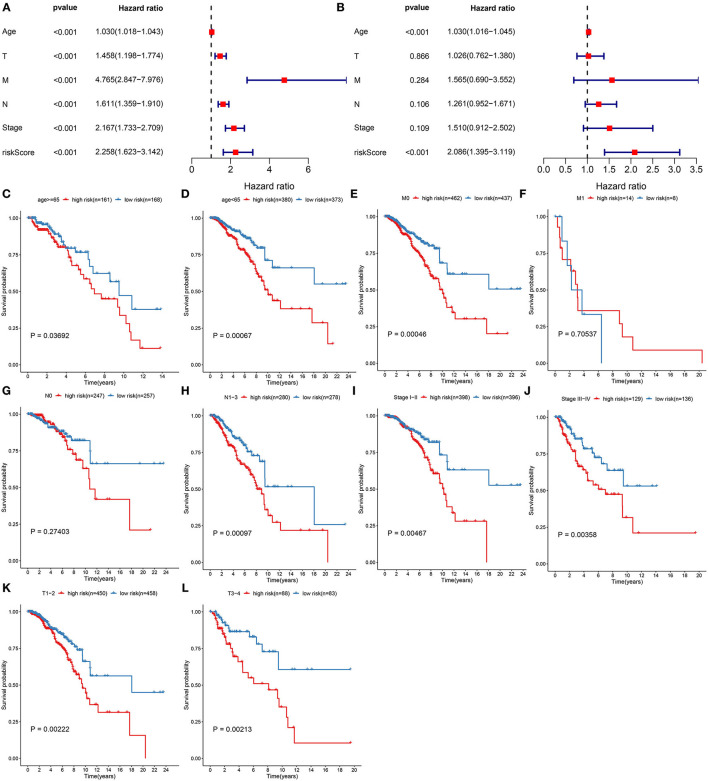
Assessment of predictive independency of the pyroptosis-related gene signature in breast cancer prognoses. **(A)** Univariate and **(B)** multivariate analyses for estimating risk scores and age, T, N, M, and stage. Subgroup analysis for investigating the differences in OS probabilities between high- and low-risk samples in **(C)** age ≥65, **(D)** age <65, **(E)** M0, **(F)** M1, **(G)** N0, **(H)** N1-3, **(I)** stage I-II, **(J)** stage III-IV, **(K)** T1-2, **(L)** T3-4 subgroups.

Correlation between clinical features and prognoses of breast cancer was analyzed via univariate analyses. In Figure 4A, age [*p* < *0.001*, HR(95%CI): 1.030(1.018–1.043)], stage [*p* < *0.001*, HR(95%CI): 2.167(1.733–2.709)], T [*p* < *0.001*, HR(95%CI): 1.458(1. 198–1.774)], *N* [*p* < *0.001*, HR(95%CI): 1.611(1.359–1.910)], M [*p* < *0.001*, HR(95%CI): 4.765(2.847–7.976)], and RS [*p* < *0.001*, HR(95%CI): 2.258(1.623–3.142)] were all risk factors of breast cancer prognoses. To investigate the independency in predicting prognoses, multivariate cox regression analyses were carried out. As a result, age [*p* < *0.001*, HR(95%CI): 1.030(1.016–1.045)] and RS [*p* < *0.001*, HR(95%CI): 2.086(1.395–3.119)] were independent risk factors (Figure 4B). Subgroup analyses were also presented for analyzing predictive sensitivity of RS. Here, all patients were stratified into different groups. Our data revealed that high RS was indicative of poorer survival outcomes in comparison to low RS in age ≥65 (*p* = *0.03692*; Figure 4C) and <65 (*p* = *0.00067*; Figure 4D), M0 (*p* = *0.00046*; Figure 4E) and M1 (*p* = *0.70537*; Figure 4F), N0 (*p* = *0.27403*; Figure 4G) and N1-3 (*p* = *0.00097*; Figure 4H), stage I-II (*p* = *0.00467*; Figure 4I) and stage III-IV (*p* = *0.00358*; Figure 4J), T1-2 (*p* = *0.00222*; Figure 4K) and T3-4 (*p* = *0.00213*; Figure 4L) subgroups.

### The Pyroptosis-Related Gene Signature Predicts Progression and Metastasis of Breast Cancer

**Figure 5:**
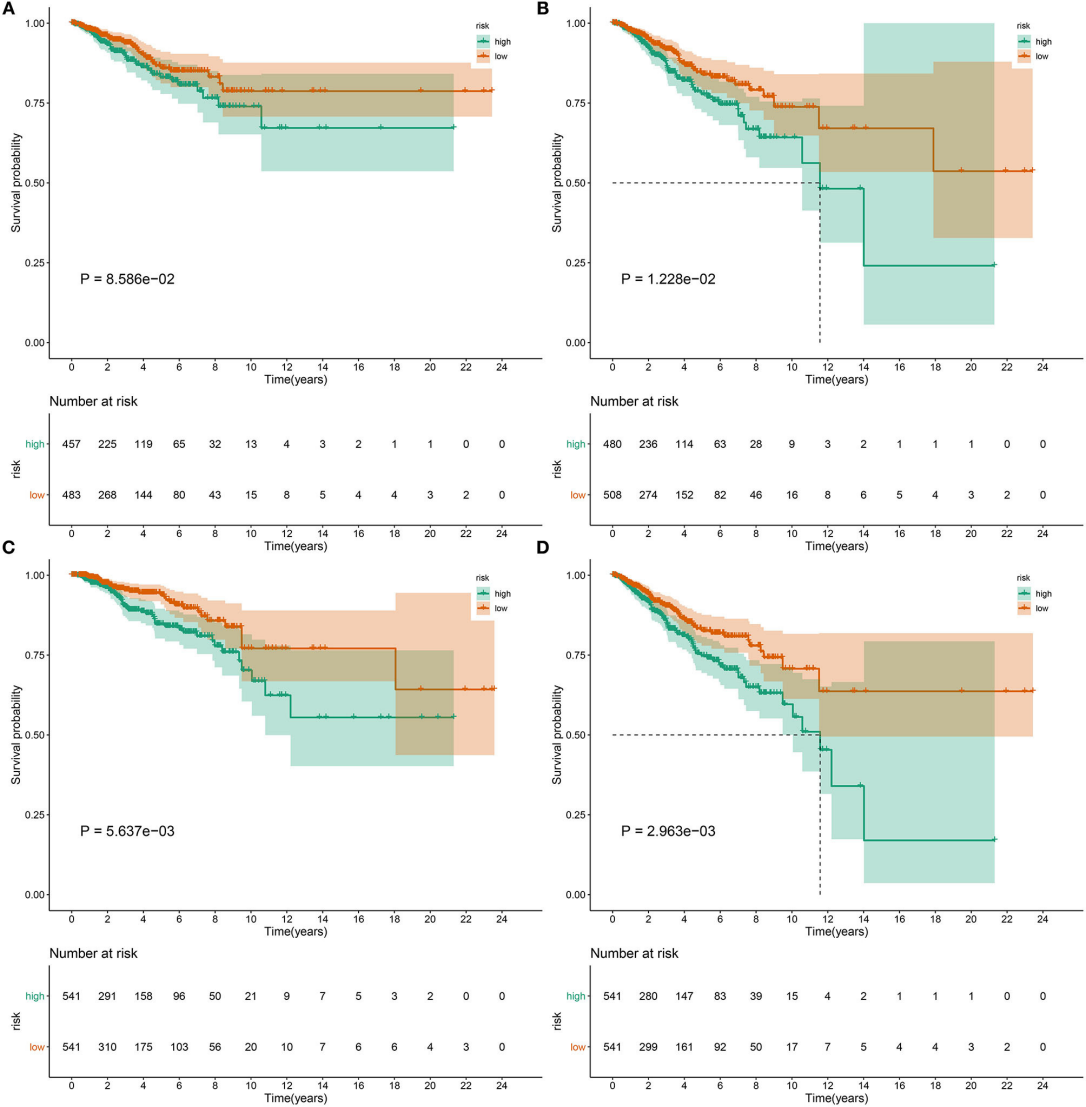
The pyroptosis-related gene signature could predict progression and metastases of breast cancer. Differences in **(A)** DFI, **(B)** DFS, **(C)** DSS, and **(D)** PFI were compared in high- and low-risk samples by Kaplan-Meier curves and log-rank tests.

This study further investigated whether the pyroptosis-related gene signature might be predictive of progression and metastasis of breast cancer. Our results demonstrated that patients with high RS displayed worse DFI (*p* = *8.586e-02*; Figure 5A), DFS (*p* = *1.228e-02*; Figure 5B), DSS (*p* = *5.637e-03*; Figure 5C) and PFI (*p* = *2.963e-03*; Figure 5D) in comparison to those with low RS.

### Association Between the Pyroptosis-Related Gene Signature and Immunogenicity

**Figure 6:**
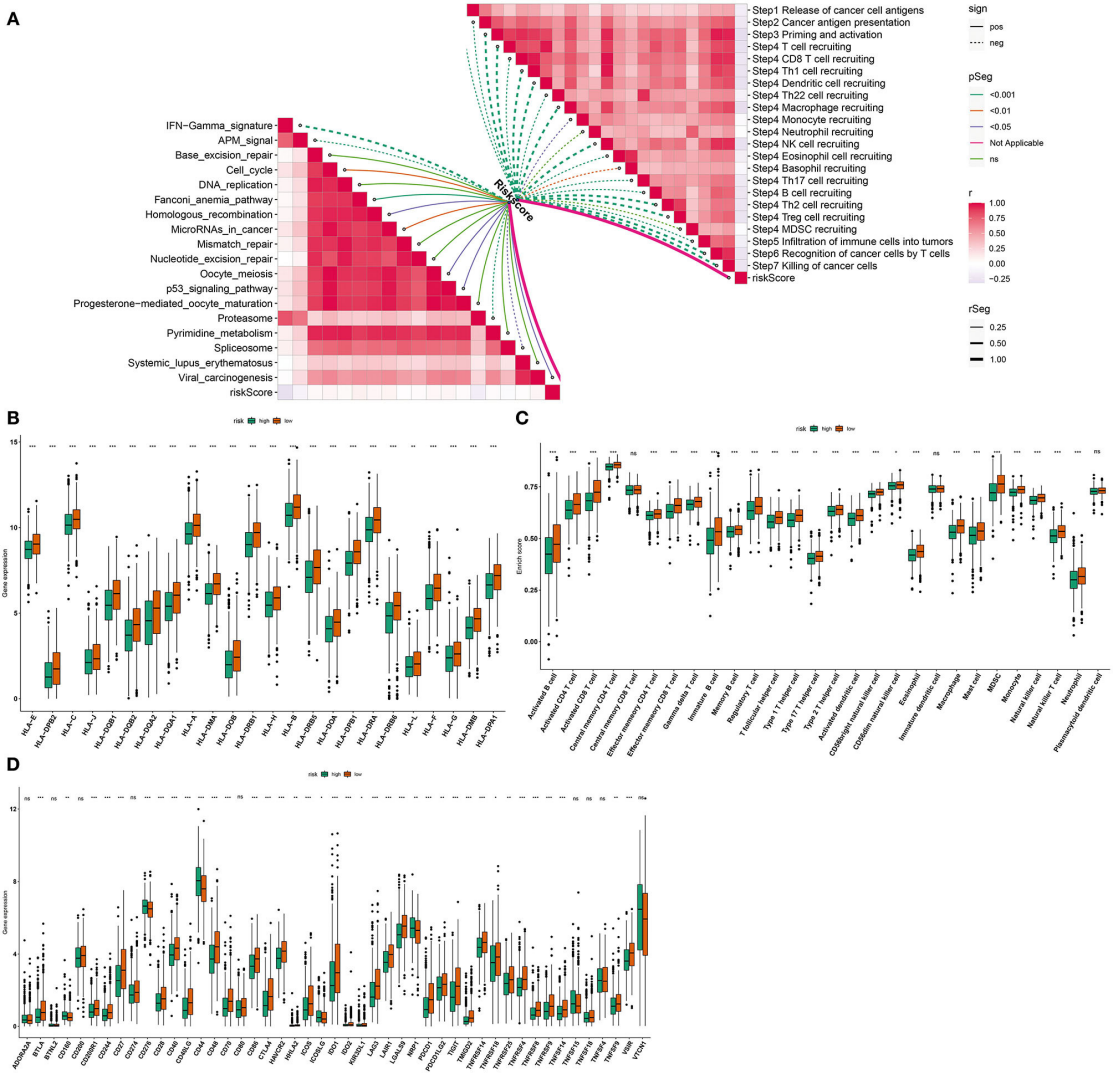
Associations between the pyroptosis-related gene signature and immunogenicity of breast cancer. **(A)** Correlations between risk score and cancer immunity cycle and pathways. Dotted line indicates negative correlation and solid line indicates positive correlation. The darker the color, the stronger the correlation. **(B)** Box plots were plotted for HLA expression in high- and low-risk samples. **(C)** Box plots were depicted to show infiltration levels of immune cells in high- and low-risk samples. **(D)** Box plots were utilized for visualizing expression of immune checkpoints in high- and low-risk samples. Ns: not significant;^*^p < *0.05;* ^**^p < *0.01;* ^***^p < *0.001* that were derived from adjusted *p*-values.

We further assessed whether the pyroptosis-related gene signature affected cancer immunity cycle. As a result, we found that the risk score almost negatively participated in each step of cancer immunity cycle, including release of cancer cell antigens, cancer antigen presentation, priming and activation, T cell recruiting, Th1 cell recruiting, dendritic cell recruiting, Th22 cell recruiting, macrophage recruiting, monocyte recruiting, neutrophil recruiting, NK cell recruiting, eosinophil cell recruiting, basophil recruiting, Th17 cell recruiting, B cell recruiting, Th2 cell recruiting, Treg cell recruiting, infiltration of immune cells into tumors, recognition of cancer cells by T cells, and killing of cancer cells (Figure 6A). Also, RS was distinctly related to the activation of IFN-gamma signature, APM signal, cell cycle, Fanconi anemia pathway, homologous recombination, microRNAs in cancer, oocyte meiosis, P53 signaling pathway, proteasome, spliceosome, and viral carcinogenesis (Figure 6A). The low RS breast cancer samples were characterized by increased expression of human lymphocyte antigen (HLA), as follows: *HLA-E, HLA-DPB2, HLA-C, HLA-J, HLA-DQB1, HLA-DQB2, HLA-DQA2, HLA-DQA1, HLA-A, HLA-DMA, HLA-DOB, HLA-DRB1, HLA-H, HLA-B, HLA-DRB5, HLA-DOA, HLA-DPB1, HLA-DRA, HLA-DRB6, HLA-L, HLA-F, HLA-G, HLA-DMB*, and *HLA-DPA1* (Figure 6B). Also, higher infiltration levels of nearly all immune cells were detected in the low RS group than the high RS group, including activated B cell, activated CD4 T cell, activated CD8 T cell, central memory CD4 T cell, effector memory CD4 T cell, effector memory CD8 T cell, gamma delta T cell, immature B cell, memory B cell, regulatory T cell, T follicular helper cell, type 1 T helper cell, type 17 T helper cell, type 2 T helper cell, activated dendritic cell, CD56bright natural killer cell, CD56dim natural killer cell, eosinophil, immature dendritic cell, macrophage, mast cell, MDSC, monocyte, natural killer cell, natural killer T cell, and neutrophil (Figure 6C). Moreover, we compared the expression of immune checkpoints between high and low RS groups. In Figure 6D, higher expression of *BTLA, CD200R1, CD244, CD27, CD28, CD40, CD40LG, CD48, CD70, CD86, CTLA4, HAVCR2, HHLA2, ICOS, IDO1, IDO2, KIR3DL1, LAG3, LAIR1, LGALS9, PDCD1, PDCD1LG2, TIGIT, TMIGD2, TNFRSF14, TNFRSF18, TNFRSF25, TNFRSF4, TNFRSF8, TNFRSF9, TNFSF14, TNFSF9*, and *VSIR* was found in the low RS group. Meanwhile, the high RS group was characterized by increased expression of *CD160, CD276, CD44, ICOSLG*, and *NRP1*. Above data suggested that RS was related to suppressive immunity in breast cancer.

### Pathways Involved in the Pyroptosis-Related Gene Signature

**Figure 7:**
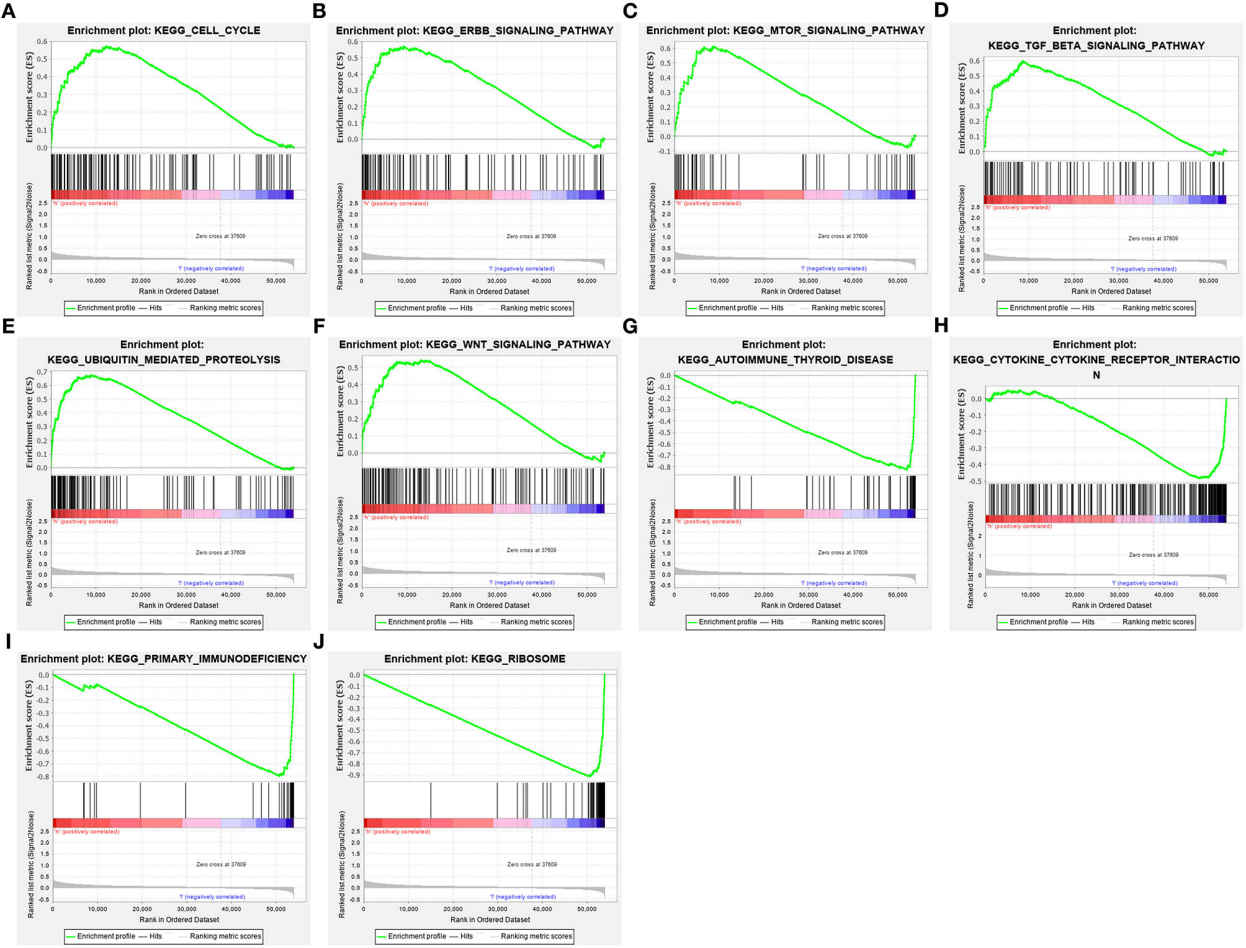
Activated pathways involved in the pyroptosis-related gene signature by GSEA. **(A)** Cell cycle, **(B)** ERBB signaling pathway, **(C)** mTOR signaling pathway, **(D)** TGF-beta signaling pathway, **(E)** ubiquitin mediated proteolysis and **(F)** WNT signaling pathway were activated in high-risk samples. **(G)** Autoimmune thyroid disease, **(H)** cytokine-cytokine receptor interaction, **(I)** primary immunodeficiency, and **(J)** ribosome were activated in low-risk samples.

GSEA was employed for exploring pathways involved in the pyroptosis-related gene signature. In Figures 7A–F, high RS was distinctly associated with cell cycle (NES = 1.85 and FDR = 0.019), ERBB signaling pathway (NES = 2.06 and FDR = 0.005), mTOR signaling pathway (NES = 2.06 and FDR = 0.004), TGF-beta signaling pathway (NES = 2.06 and FDR = 0.005), ubiquitin mediated proteolysis (NES = 2.22 and FDR = 0.002) and WNT signaling pathway (NES = 2.09 and FDR = 0.005). Meanwhile, low RS was in relation to autoimmune thyroid disease (NES = −2.24 and FDR = 0.001), cytokine-cytokine receptor interaction (NES = −1.74 and FDR = 0.035), primary immunodeficiency (NES = −1.95 and FDR = 0.007), and ribosome (NES = −2.13 and FDR = 0.003; Figures 7G–J).

### Development of a Prognostic Nomogram for Breast Cancer

**Figure 8:**
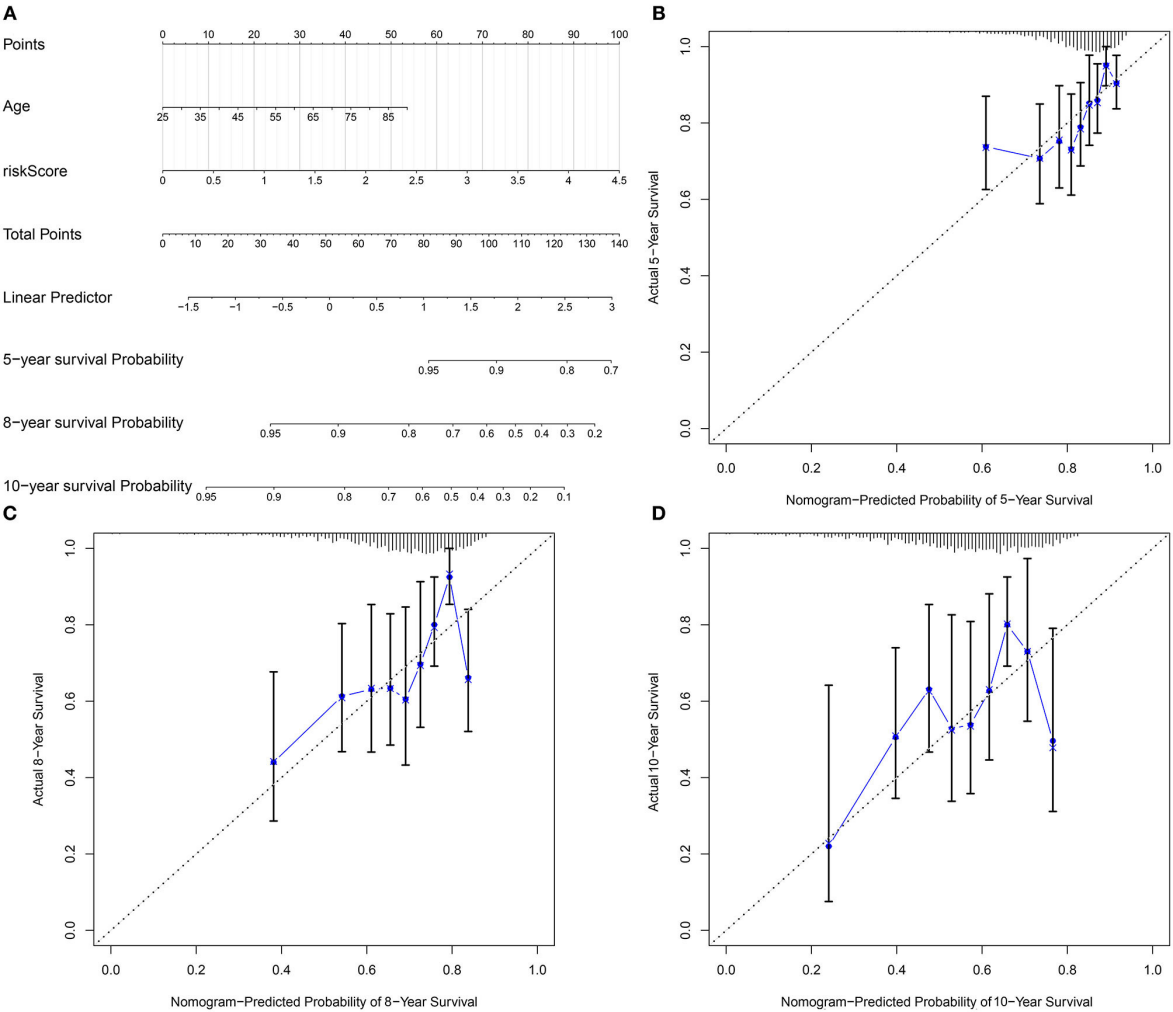
Development of a nomogram for estimating 5-, 8-, and 10-year survival probabilities of breast cancer patients. **(A)** A nomogram incorporating age and risk score was a predictor of 5-, 8-, and 10-year survival probabilities. **(B–D)** Calibrate plots was applied for investigating the deviation in nomogram-predicted and actual 5-, 8-, and 10-year survival probabilities.

Our multivariate analyses demonstrated that age and the pyroptosis-related gene signature were independent risk factors of breast cancer, which were used for developing a prognostic nomogram. In Figure 8A, this nomogram could predict 5-, 8- and 10-year survival probabilities. The predictive performance was evaluated by calibration plots. There were high consistencies in nomogram-predicted and actual 5-, 8- and 10-year survival (Figures 8B–D). Our data suggested that the nomogram exhibited the well predictive efficacy in 5-, 8- and 10-year survival probabilities.

### Validation of the Expression of Genes in the Pyroptosis-Related Gene Signature

**Figure 9:**
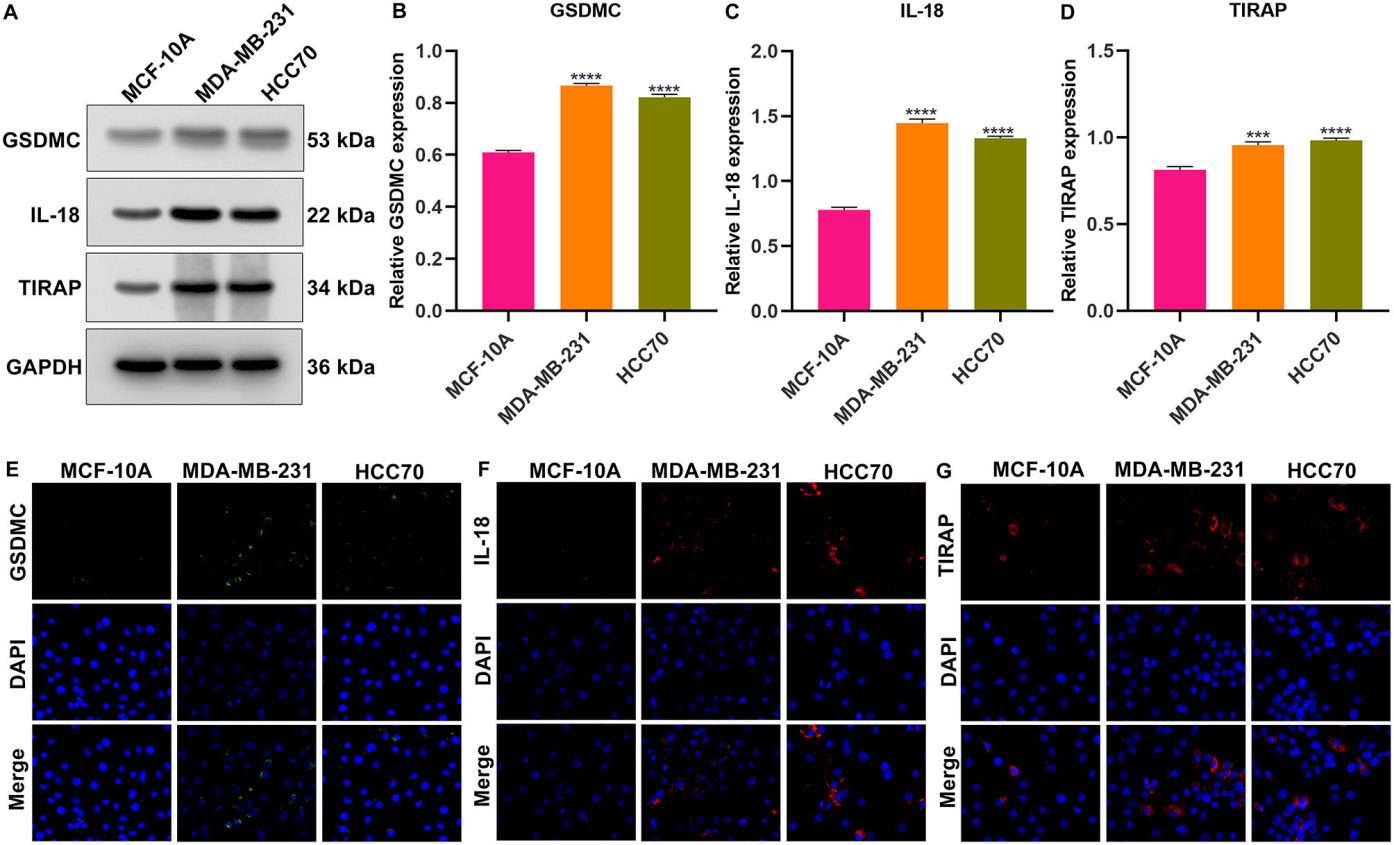
Validation of the expression of *GSDMC, IL-18*, and *TIRAP* in normal breast cells (MCF-10A) and breast cancer cells (MDA-MB-231 and HCC70). **(A–D)** Western blot detecting the expression of GSDMC, IL-18, and TIRAP in MCF-10A, MDA-MB-231, and HCC70 cells. ^***^p < *0.001;* ^****^p < *0.0001*. **(E–G)** Immunofluorescence of the expression of GSDMC, IL-18, and TIRAP in MCF-10A, MDA-MB-231, and HCC70 cells. Scale bar, 5 μm; magnification 200 ×.

We further validated the expression of *IL-18, GSDMC*, and *TIRAP* in the pyroptosis-related gene signature *in vitro*. As depicted in western blotting, *IL-18, GSDMC*, and *TIRAP* expression was all markedly up-regulated in breast cancer cells MDA-MB-231 and HCC70 compared with normal breast cells MCF-10A (Figures 9A–D). Immunofluorescence also confirmed the significant up-regulation of *IL-18, GSDMC* and *TIRAP* expression in MDA-MB-231 and HCC70 cells than MCF-10A cells (Figures 9E–G).

## Discussion

Programmed cell fate nearly focuses on apoptosis and necroptosis. It is of importance to find an alternative option when these cell deaths are compromised (26). Pyroptotic death represents a form of programmed cell death, which is induced by inflammasomes (27). Inducing apoptosis of cancer cells has been applied for eliminating malignant cells (28). Nevertheless, due to escaping apoptosis, induction of pyroptosis may be especially critical in treating antiapoptotic cancers. Immunotherapies show remarkable efficacy in treating breast cancer. Nevertheless, therapeutic effects are still limited (29). Pyroptosis offers an opportunity for alleviating immunosuppression as well as promoting an immune response in treating breast cancer (30). Here, we characterized expression, genetic mutations, and clinical implications of pyroptosis-related genes in breast cancer.

Among pyroptosis-related genes, *GPX4, NLRP7, CASP6, CASP3, IL1B, IL18, CASP8, NLRP6, IL6, GSDMC, PYCARD, AIM2, NOD2, NLRP3*, and *CASP4* were aberrantly expressed in breast cancer. Furthermore, *CASP8* (2%), *NLRC4* (1%), NLRP3 (1%), *NLRP2* (1%), *PLCG1* (1%), *NLRP1* (1%), *NLRP7* (1%), *SCAF11* (1%), *GSDMC* (1%), and *NOD1* (1%) occurred somatic mutations as well as most of them had high frequencies of CNV in breast cancer. Our spearman test and PPI network both revealed the tight interactions between pyroptosis-related genes. In the PPI network, *CASP1* had the highest degree. Consistently, *CASP1* is a prognostic factor as well as therapeutic target in breast cancer (31). As shown in GO enrichment analyses, pyroptosis-related genes were primarily involved in mediating *IL-1*β production and secretion biological processes as well as cytosolic, inflammasome and membrane cellular components and apoptotic process, indicating the key biological implications of pyroptosis in tumorigenesis. We generated a pyroptosis-related gene signature, containing *IL-18, GSDMC*, and *TIRAP*. High RS was indicative of undesirable OS, recurrence, and progression of breast cancer. AUC = 0.652 demonstrated the well predictive efficacy. Multivariate analyses and subgroup analyses suggested that this RS was an independent risk factor of breast cancer prognoses. Previously, a pyroptosis-related gene signature was generated for prediction of ovarian cancer prognoses (14). Our univariate cox regression analyses showed that *IL-18* was a protective factor as well as *GSDMC* and *TIRAP* were risk factors for breast cancer prognoses. *IL-18*, a proinflammatory cytokine, modulates inflammation, and immune response. As confirmed by previous studies, mesenchymal stem cells expressing *IL-18* suppresses breast cancer proliferation and metastasis, suggesting the antitumor activities of *IL-18* (32, 33). *GSDMC* is specifically cleaved by *caspase-8* through *TNF*α to generate *GSDMC* N-terminal domain, thereby forming pores on the cell membrane as well as inducing pyroptosis. High expression of *GSDMC* is in relation to undesirable survival of cancer patients (34). Furthermore, there is evidence shows that *TIRAP* is a risk factor of cancer prognosis (35).

Immunotherapies especially immune checkpoint inhibitors (ICIs) may produce durable therapeutic effects. Nevertheless, only one third of patients respond to ICIs. Breast cancer is often considered as a cold tumor, with lowly frequent mutation, decreased immune cell infiltration, and suppressive immune microenvironment (36). Inducing cell deaths other than apoptosis has been considered as novel cancer therapeutic strategies due to innate resistance to apoptosis (37). Combination of inducing pyroptosis and ICIs could display synergistically increased anti-cancer activity (28, 38, 39). However, most evidences of interaction between immunity and pyroptosis are derived from animal and cellular model. Here, pyroptosis-related gene signature was negatively correlated to almost all steps of cancer immunity cycle (40). Also, we found that low RS was characterized by high HLA, immune cell infiltrations and immune checkpoints. This suggested that pyroptosis exerted an impact on immuno-oncology.

In-depth exploring pyroptosis mechanisms, up- and downstream pathways may offer novel insights into breast cancer therapy. Our GSEA results suggested that high RS was in relation to carcinogenic pathways including cell cycle, ERBB signaling pathway, mTOR signaling pathway, TGF-beta signaling pathway, ubiquitin mediated proteolysis and WNT signaling pathway. Also, low RS was significantly related to immune-related pathways including autoimmune thyroid disease, cytokine-cytokine receptor interaction, primary immunodeficiency, and ribosome. These data indicated the interactions of pyroptosis-relevant RS with above pathways in breast cancer progression. Epidemiologic studies have confirmed that age is a risk factor of breast cancer (41). Personalized medicine is based on individual evaluation of risk. By including two independent risk factors age and this RS, we generated a nomogram for prediction of 5-, 8-, and 10-year survival probabilities. The predictive accuracy was confirmed by comparing observed survival duration.

There are several limitations in our study. Firstly, due to the limited clinical features of patients, we cannot carry out subgroup analyses by stratifying more factors. Secondly, the pyroptosis-relevant RS was constructed and verified based on retrospective cohorts. In conclusion, our findings revealed that pyroptosis induction might be a novel strategy for breast cancer immunotherapy, characterized by high compatibility and extensive clinical applicability. In future studies, prognostic implications of pyroptosis will be observed in a larger breast cancer cohort. Also, interactions of pyroptosis with cancer immunity will be further verified in cellular and animal models.

## Conclusion

Collectively, our data characterized expression patterns and mutations of pyroptosis-related genes. A three-gene regression model including *IL18, GSDMC*, and *TIRAP* was regarded as an independent risk factor of breast cancer prognoses. The RS was distinctly cancer immunity cycle, HLA immune cell infiltration and immune checkpoints in breast cancer. Our data suggested that pyroptosis combining with immunotherapies might be a potential therapeutic strategy.

## Data Availability Statement

The original contributions presented in the study are included in the article/Supplementary Material, further inquiries can be directed to the corresponding author.

## Author Contributions

LQ conceived and designed the study. DX conducted most of the experiments and data analysis, and wrote the manuscript. ZJ participated in collecting data and helped to draft the manuscript. All authors reviewed and approved the manuscript.

## Conflict of Interest

The authors declare that the research was conducted in the absence of any commercial or financial relationships that could be construed as a potential conflict of interest.

## Publisher's Note

All claims expressed in this article are solely those of the authors and do not necessarily represent those of their affiliated organizations, or those of the publisher, the editors and the reviewers. Any product that may be evaluated in this article, or claim that may be made by its manufacturer, is not guaranteed or endorsed by the publisher.

## Supplementary Material

Supplementary Table 1 Clinical information of 1,082 breast cancer patients.

Supplementary Table 2 The gene sets of cancer-related pathways.

## Abbreviations

ICIs: immune checkpoint inhibitors
TCGA: the Cancer Genome Atlas
CNV: copy number variation
PPI: protein-protein interaction
GO: Gene ontology
RS: risk score
ROC: receiver operating characteristic
HR: hazard ratio
CI: confidence interval
DFI: disease-free interval
DFS: disease-free survival
DSS: disease-specific survival
PFI: progression -free interval
ssGSEA: single sample gene set enrichment analysis
GSEA: Gene set enrichment analysis
KEGG: Kyoto Encyclopedia of Genes and Genomes.
