# Supplementary figures and images for: Molecular Characteristics, Clinical Implication, and Cancer Immunity Interactions of Pyroptosis-Related Genes in Breast Cancer
Source: Front Med (Lausanne). 2021 Sep 13;8:702638. doi: 10.3389/fmed.2021.702638 (PMC8473741; doi:10.3389/fmed.2021.702638)

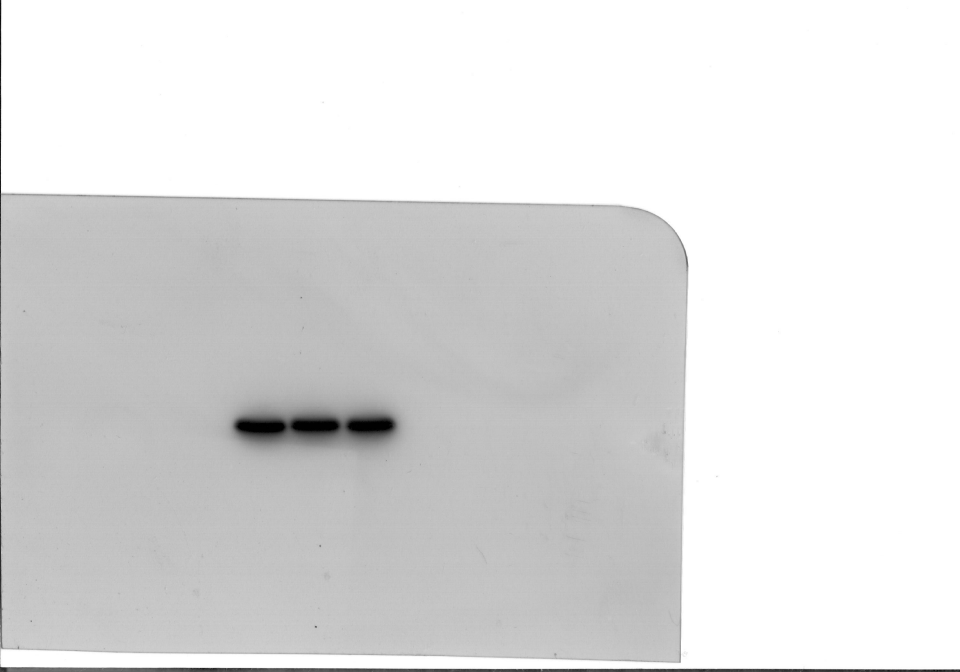

Supplement: Supplementary file 3 [file Data_Sheet_1.ZIP › Original Source Data/Figure 9/Figure 9A/GAPDH.pdf]

000(YTETHAS)

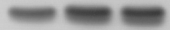

Supplement: Supplementary file 3 [file Data_Sheet_1.ZIP › Original Source Data/Figure 9/Figure 9A/GSDMC.pdf]

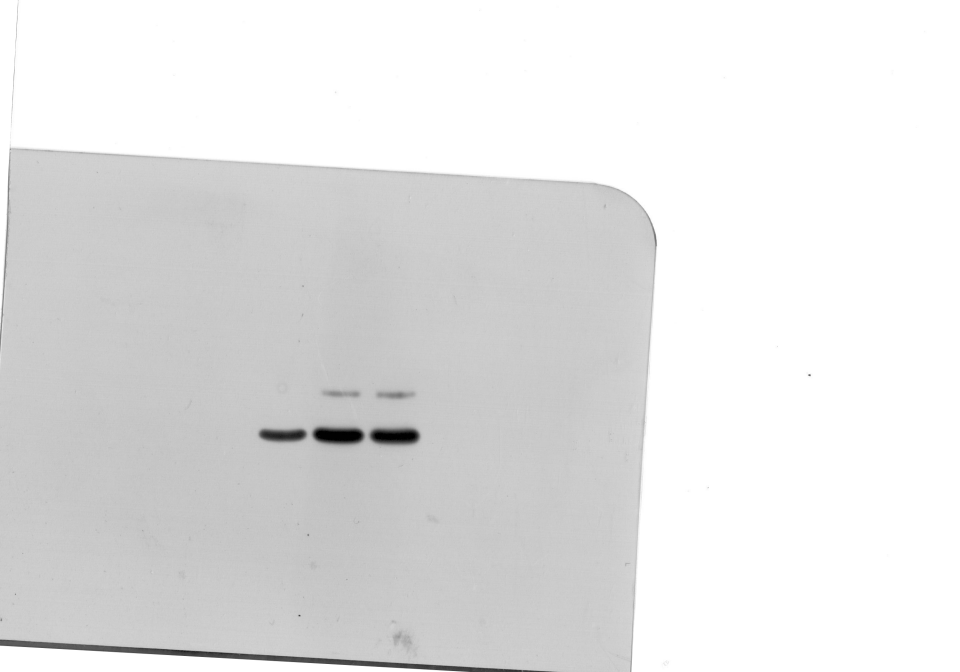

Supplement: Supplementary file 3 [file Data_Sheet_1.ZIP › Original Source Data/Figure 9/Figure 9A/IL-18.pdf]

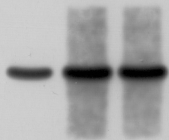

Supplement: Supplementary file 3 [file Data_Sheet_1.ZIP › Original Source Data/Figure 9/Figure 9A/TIRAP.pdf]

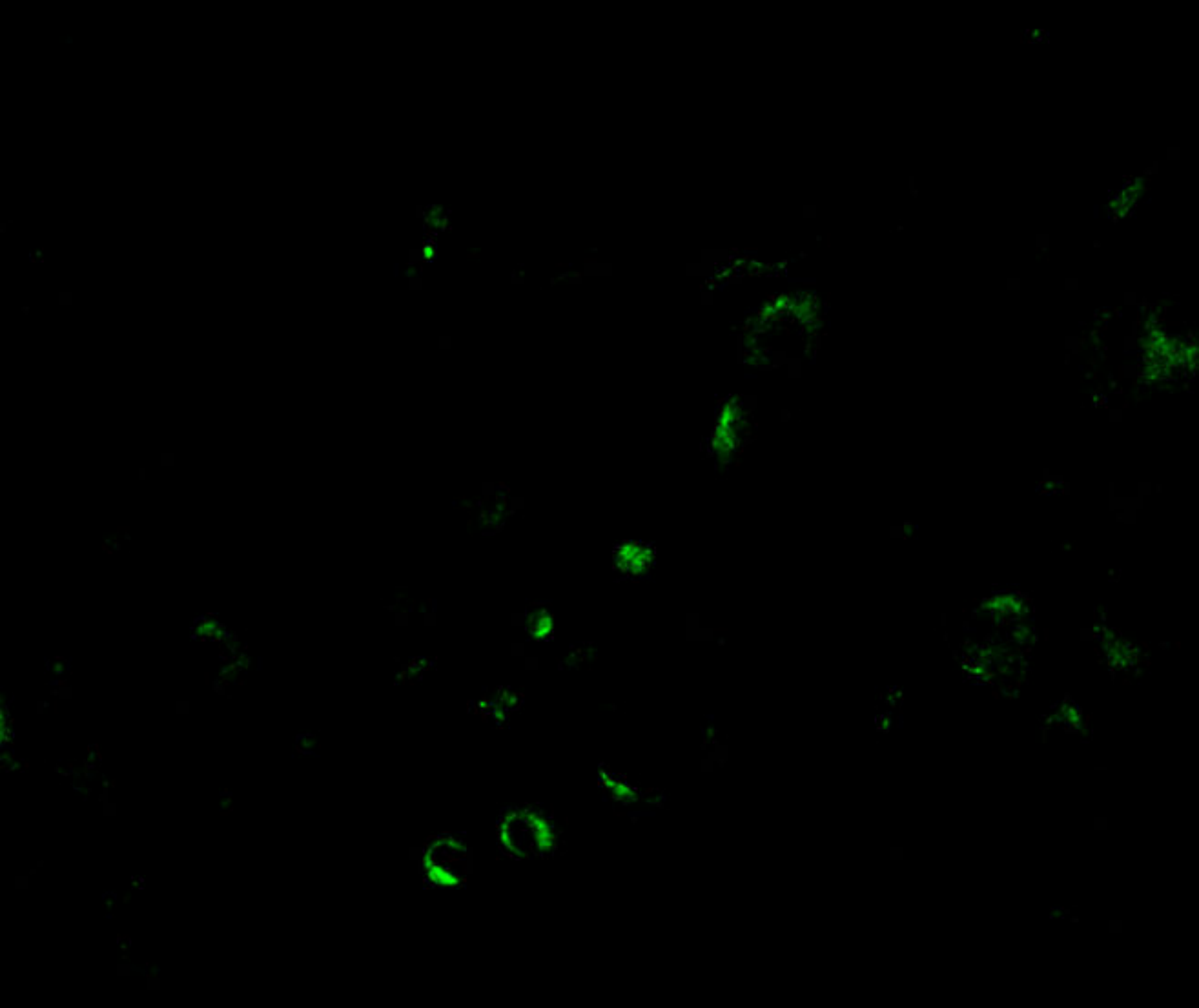

Supplement: Supplementary file 3 [file Data_Sheet_1.ZIP › Original Source Data/Figure 9/Figure 9E/MDA-MB-231-GSDMC.pdf]

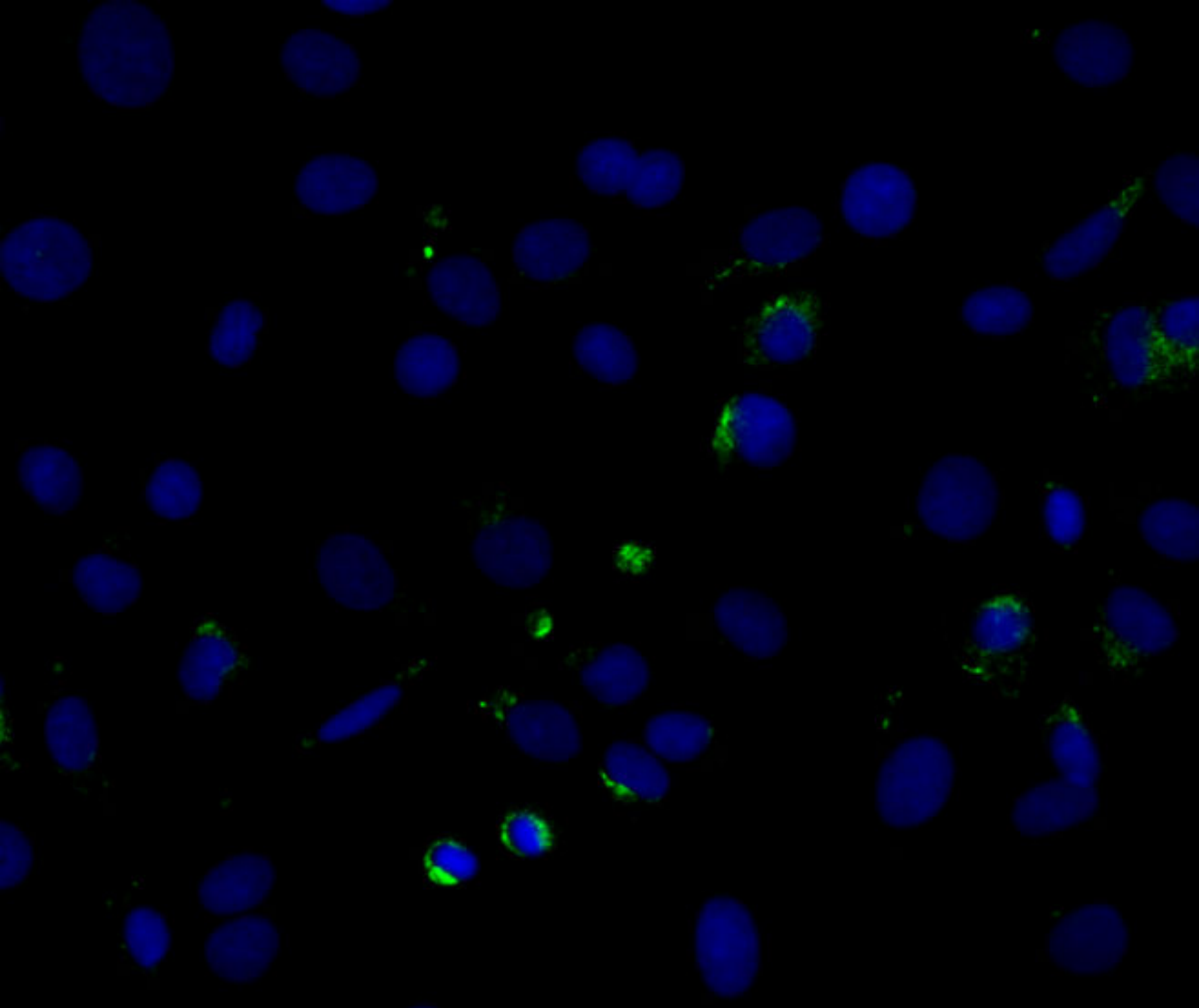

Supplement: Supplementary file 3 [file Data_Sheet_1.ZIP › Original Source Data/Figure 9/Figure 9E/MDA-MB-Merge.pdf]

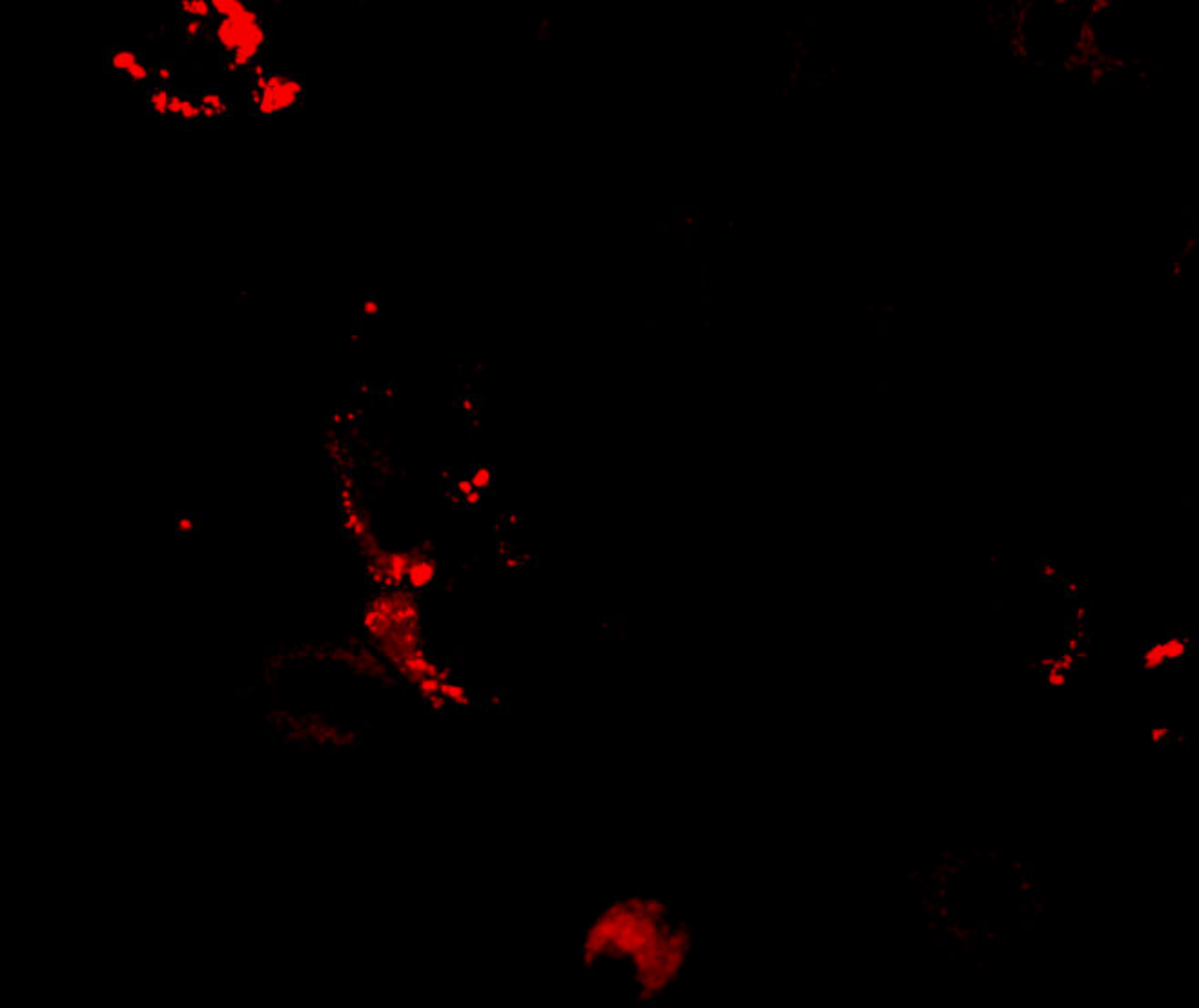

Supplement: Supplementary file 3 [file Data_Sheet_1.ZIP › Original Source Data/Figure 9/Figure 9F/HCC70-IL-18.pdf]

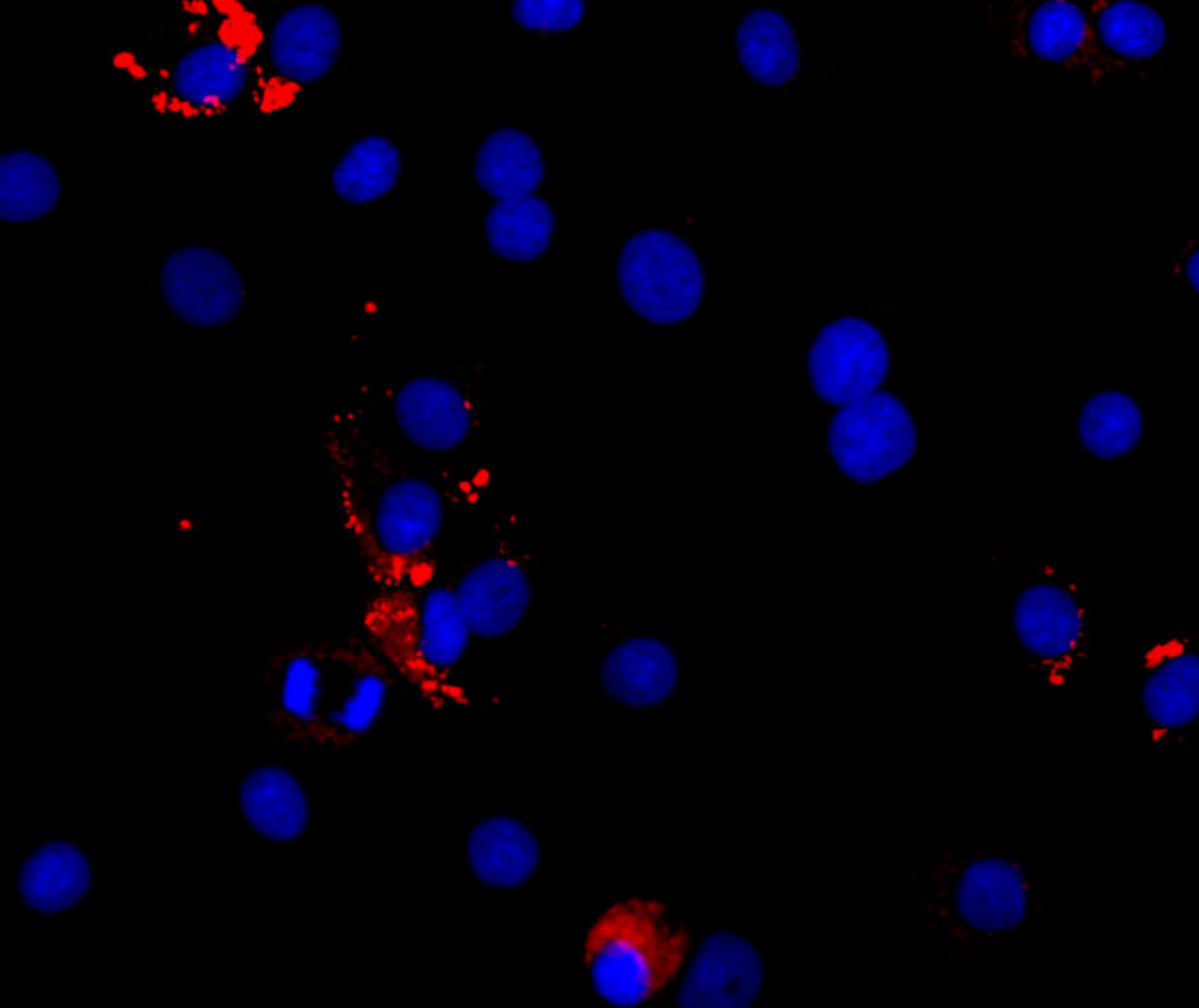

Supplement: Supplementary file 3 [file Data_Sheet_1.ZIP › Original Source Data/Figure 9/Figure 9F/HCC70-Merge.pdf]

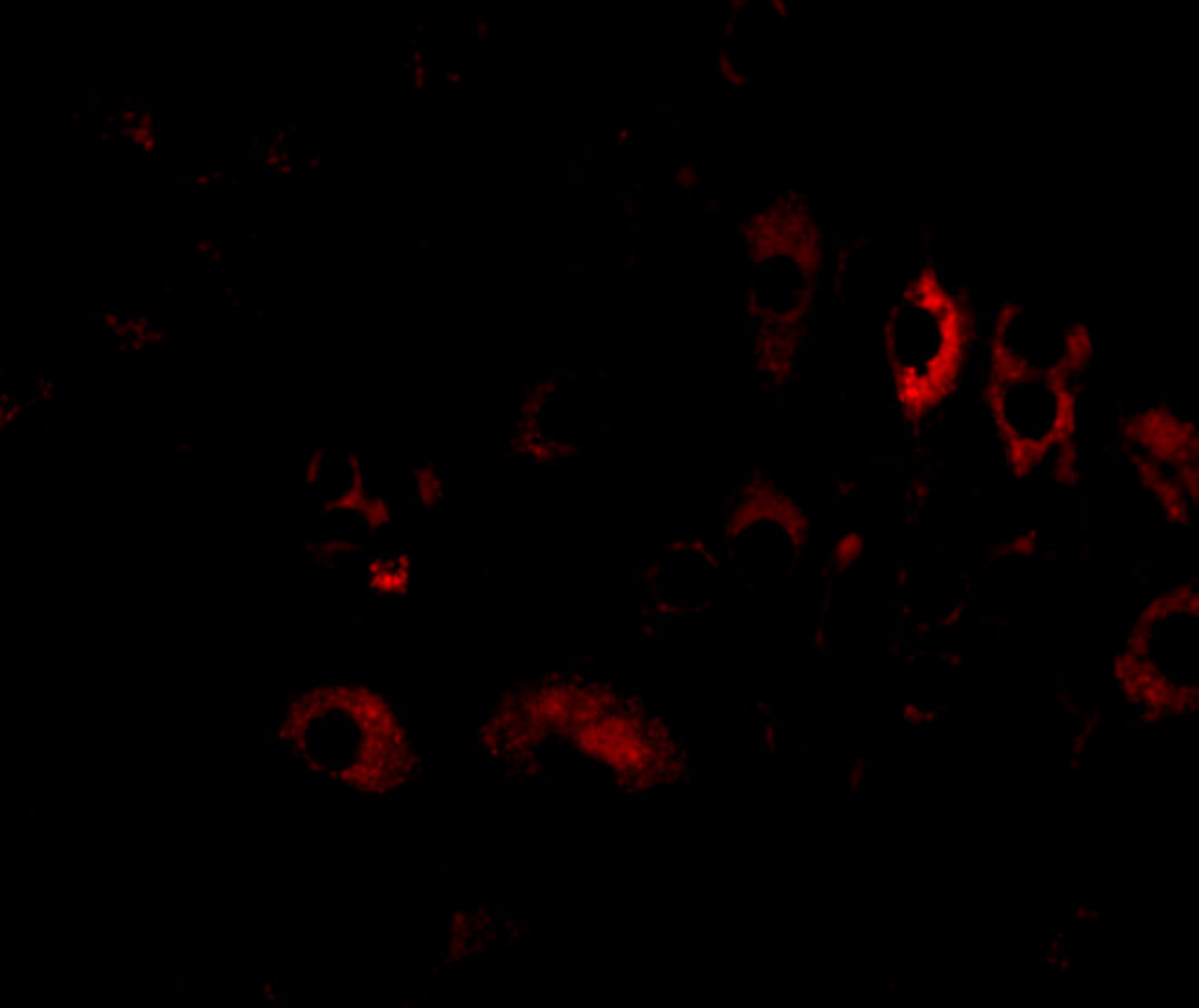

Supplement: Supplementary file 3 [file Data_Sheet_1.ZIP › Original Source Data/Figure 9/Figure 9G/MDA-MB-231-TIRAP.pdf]
