# Supplementary material for: Molecular Characteristics, Clinical Implication, and Cancer Immunity Interactions of Pyroptosis-Related Genes in Breast Cancer
Source: Front Med (Lausanne). 2021 Sep 13;8:702638. doi: 10.3389/fmed.2021.702638 (PMC8473741; doi:10.3389/fmed.2021.702638)

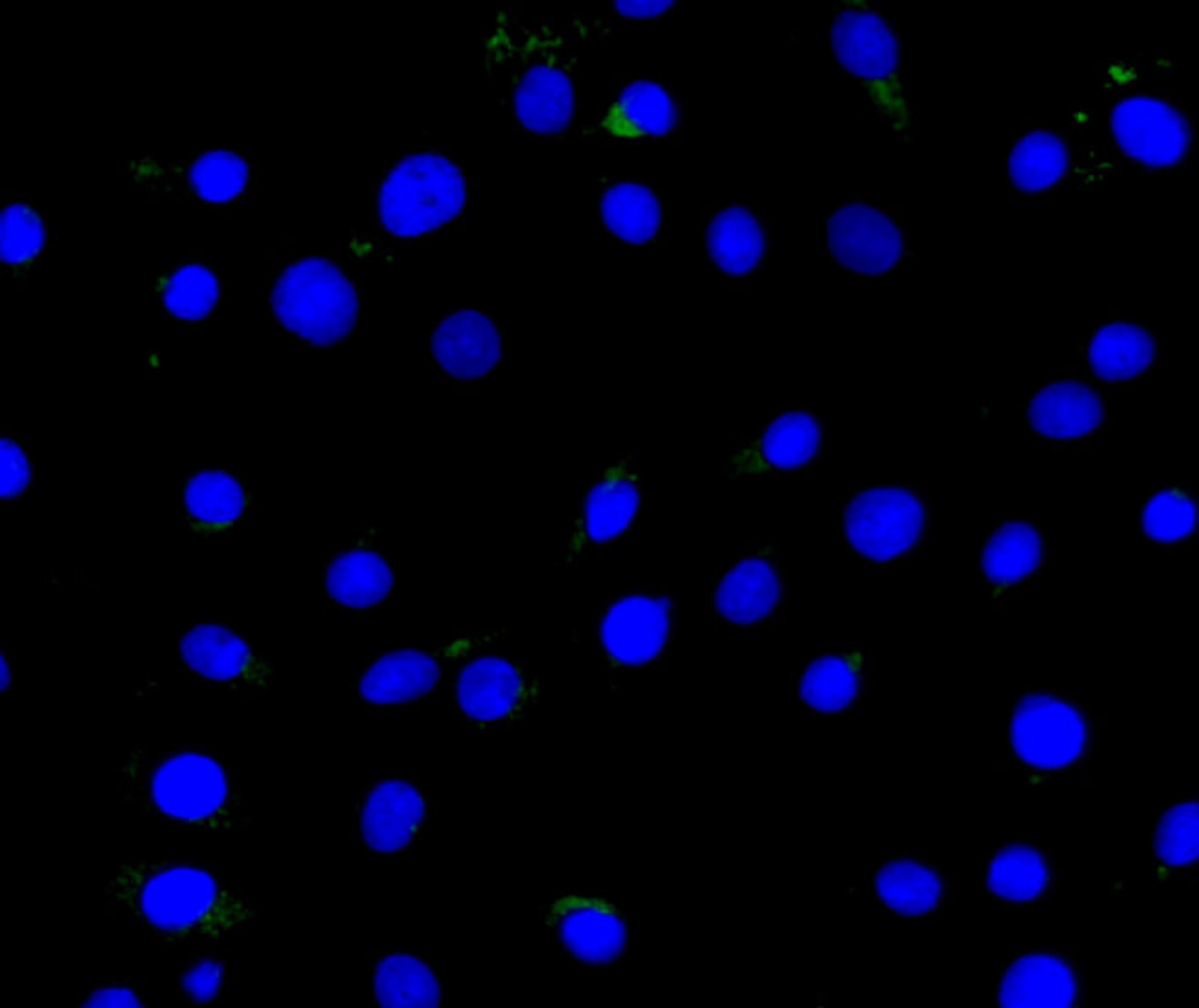

Supplement: Supplementary file 3 [file HCC70-Merge.pdf › Original Source Data/Figure 9/Figure 9E/HCC70-Merge.pdf]
